# The Diseases of Women

**Published:** 1898-06

**Authors:** 


**The Diseases of Women.** By J. BLAND SUTTON and ARTHUR E. GILES, M.D. Pp. 436. London: The Rebman Publishing Co., Ltd. Philadelphia: W. B. Saunders. 1897.

Although works on this subject have so largely increased of late, we welcome the present volume, which gives a clear and concise account of the matter of which it treats. Indeed we think that the pity is, there is not more of it. It is too brief. This is partly owing to the fact that too little mention is made of methods other than those which the authors think the best. For example, in the treatment of fibroids by hysterectomy the extra-peritoneal treatment of the pedicle is absolutely ignored. This may be considered up to date, but it is doubtful whether it is always best in surgery to merely inculcate the ideal methods without referring to what may at least have to be done of necessity if not of choice. Much of the pathology and many of the excellent illustrations will be recognised by those familiar with the writings of one of the authors. We should much like to see a future edition assume considerably larger proportions, for we feel that the subject is in able hands.

